# A High Lymph Node Yield is Associated with Prolonged Survival in Elderly Patients Undergoing Curative Gastrectomy for Cancer: A Dutch Population-Based Cohort Study

**DOI:** 10.1245/s10434-017-5815-5

**Published:** 2017-02-28

**Authors:** Hylke J. F. Brenkman, Lucas Goense, Lodewijk A. Brosens, Nadia Haj Mohammad, Frank P. Vleggaar, Jelle P. Ruurda, Richard van Hillegersberg

**Affiliations:** 1 Department of Surgery, University Medical Center Utrecht, Utrecht, The Netherlands; 2 Department of Radiation Oncology, University Medical Center Utrecht, Utrecht, The Netherlands; 3 Department of Pathology, University Medical Center Utrecht, Utrecht, The Netherlands; 4 Department of Medical Oncology, University Medical Center Utrecht, Utrecht, The Netherlands; 5 Department of Gastroenterology and Hepatology, University Medical Center Utrecht, Utrecht, The Netherlands

## Abstract

**Purpose:**

The aim of this study was to evaluate the influence of lymph node yield (LNY) on postoperative mortality and overall survival in elderly patients with gastric cancer.

**Methods:**

This population-based study included data from The Netherlands Cancer Registry of patients who underwent curative gastrectomy for adenocarcinoma between 2006 and 2014. Patients were divided into two groups based on age (<75 years, young; ≥75 years, elderly). LNY was analyzed as both a categorical variable (low, <15 nodes; intermediate, 15–25 nodes; high, >25 nodes), and a discrete variable. Multivariable analysis was used to evaluate the influence of LNY on 30- and 90-day mortality, as well as overall survival.

**Results:**

A total of 3764 patients were included in the study; 2387 (63%) were classified as ‘young’, and 1377 (37%) were classified as ‘elderly’. The median LNY was 14 in the young group, compared with 11 in the elderly group (*p* < 0.001). In the elderly group, 851 (62%) patients had a low LNY, 333 (24%) had an intermediate LNY, and 174 (13%) had a high LNY. Multivariable analysis demonstrated that in the elderly patients, a higher LNY was associated with a prolonged overall survival (low: reference; intermediate: hazard ratio [HR] 0.74, 95% confidence interval [CI] 0.62–0.88, *p* < 0.001; high: HR 0.59, 95% CI 0.45–0.78, *p* < 0.001), but not with 30-day (*p* = 0.940) and 90-day mortality (*p* = 0.573). For young patients, these results were comparable.

**Conclusion:**

In both young and elderly patients, a high LNY is associated with prolonged survival but not with an increase in postoperative mortality. Therefore, an extensive lymphadenectomy is the preferred strategy for all patients during gastrectomy in order to provide an optimal oncological result.

Worldwide, surgical treatment of gastric adenocarcinoma consists of resection of the stomach combined with a lymphadenectomy to remove both macro- and micrometastases of the tumor.^1^ In the past, several studies have compared a D1 lymphadenectomy, including perigastric lymph nodes, with a D2 lymphadenectomy, including both perigastric lymph nodes and locoregional lymph nodes. These studies found a survival benefit of D2 lymphadenectomy over D1 lymphadenectomy.^2^,^3^ As a result, international guidelines recommend D2 lymphadenectomy for all advanced-stage tumors (cT2-4 or cN+).^4^,^5^

Elderly patients undergoing major cancer surgery are prone to postoperative morbidity and mortality due to pre-existent comorbidities.^6^ Additionally, in some studies a more extensive lymphadenectomy has been associated with higher postoperative morbidity and mortality.^7^,^8^ These short-term outcomes may be more relevant in elderly patients as the expected survival benefit from an extensive lymphadenectomy is lower compared with younger patients. As elderly patients form a substantial portion of the patients undergoing gastrectomy for cancer,^9^ the extent of lymphadenectomy in the elderly is currently under debate.

Lymph node yield (LNY) has frequently been used as a surrogate for the extent of lymphadenectomy.^10^ Therefore, the current study aimed to evaluate the influence of LNY on postoperative mortality and overall survival in both young and elderly patients with gastric cancer.

## Materials and Methods

### Study Design

This population-based observational cohort study included data from The Netherlands Cancer Registry (NCR), which has an area comprising nearly 17 million inhabitants. In The Netherlands, all newly diagnosed cancers are registered in the NCR, which is maintained by the Netherlands Comprehensive Cancer Organisation (IKNL). The National Automated Pathology Archive (PALGA) and the National Registry of Hospital Discharge Diagnoses are important sources for the NCR. Trained data managers register data from hospital records within all Dutch hospitals on a daily basis using the NCR’s registration and coding manual, and survival status is updated yearly from the civil registry. The NCR’s privacy committee approved this study.

### Patients

In this study, data from patients who underwent a curative gastrectomy for gastric adenocarcinoma (pT0-4a, N0-3, M0) during the period 2006–2014 were selected from the NCR. Patients who underwent multi-organ surgery and patients without follow-up were excluded. Data on patient and treatment-related characteristics, histopathological characteristics, and follow-up were extracted from the NCR, whereas data regarding patients’ comorbidities and postoperative morbidity were not available from the NCR.

### Diagnosis, Treatment, and Follow-Up

Patients were diagnosed and treated according to the Dutch national guidelines for the diagnosis, treatment, follow-up, and guidance of patients with gastric cancer.^11^ The diagnostic work-up consisted of endoscopy with tumor biopsy and computed tomography (CT). In most cases, patients who underwent neoadjuvant chemotherapy received a regimen comparable to epirubicin, cisplatin, and capecitabine.^12^ Since 2010, gastric surgery has been centralized in The Netherlands, aiming for a minimum of 20 procedures per center per year.^13^ Surgery consisted of a subtotal or total gastrectomy, depending on the possibility of achieving a proximal resection margin of ≥6 cm.^11^ In all patients, national guidelines recommended a D2 lymphadenectomy without station 10 dissection, pancreatectomy, and splenectomy, but the NCR did not include information on the actual lymphadenectomy performed. Resection specimens were reviewed by pathologists in accordance with the Union for International Cancer Control (UICC) TNM staging system.^14^ Tumors that were staged according to the 6th edition were translated to the 7th edition.^5^ The routine follow-up of patients consisted of medical history and physical examination at the outpatient clinic after 6 weeks, 6 months, 12 months, and yearly thereafter until discharge of follow-up after 5 years. Radiological imaging was not routinely performed during follow-up.

### Outcome Measures

All included patients were divided into two groups based on age according to a previous study;^10^ patients younger than 75 years (<75 years group, young) and patients aged 75 years or older (≥75 years group, elderly). The LNY was categorized into three groups according to a previous study:^10^ low (<15 nodes), intermediate (15–25 nodes), and high (>25 nodes) LNY. (Sub) acute surgery was defined as surgery within <7 days after diagnosis, and postoperative mortality was analyzed within 30- and 90-days after surgery. Overall survival was calculated in months from the day of surgery until death or the end of follow-up on 31 December 2015.

### Statistical Analyses

To assess the distribution of all baseline, surgical, and histopathological characteristics, a comparison was made between the three groups of LNYs (<15, 15–25, and >25 nodes). Categorical variables were analyzed using the *χ* ^2^ test and continuous variables were compared using one-way analysis of variance (ANOVA). To evaluate the influence of LNY on postoperative mortality, univariable and multivariable logistic regression analysis was performed, providing odds ratios (ORs) with 95% confidence intervals (CIs). In addition, the influence of LNY on overall survival was evaluated using univariable and multivariable Cox proportional hazards models, providing hazard ratios (HRs) along with 95% CIs. For the multivariable Cox analysis, a nonparsimonious approach was used for the selection of model variables, including all patient- and treatment-related characteristics, as well as LNY. LNY was included as both a categorical variable and a discrete variable. Results were stratified according to age (<75 and ≥75 years), and a subgroup analysis was performed based on the radicality of the resection (R0/R+). For all Cox proportional hazard models, nonviolation of the proportional hazards assumption was verified with log-minus-log plots. Adjusted survival curves were made from the proportional hazards models.

## Results

### Study Population

The NCR selected data from 3814 patients who underwent a curative gastrectomy for gastric adenocarcinoma; a total of 50 patients were excluded as a result of multi-organ surgery (*n* = 45) or lack of follow-up (*n* = 5). Of the remaining 3764 patients, 2387 (63%) were younger than 75 years of age and 1377 (37%) were aged 75 years or older. Patient and treatment-related characteristics and their comparison between young and elderly patients are presented in Table 1.

**Table 1:** Baseline characteristics of 3764 patients who underwent gastrectomy with curative intent for cancer, stratified by age (<75 and ≥75 years)

	All		Young (<75 years)		Elderly (≥75 years)		*p* value
	*n* = 3764	(%)	*n* = 2387	(%)	*n* = 1377	(%)	
Age, years (mean [SD])	68.7	[±11.8]	62.3	[9.9]	80.0	[3.8]	**<0.001**
Sex							0.058
Male	2305	(61)	1489	(62)	816	(59)	
Female	1459	(39)	898	(38)	561	(41)	
Malignancy history							**<0.001**
No	3265	(87)	2140	(90)	252	(18)	
Yes	499	(13)	247	(10)	1125	(82)	
Referral for gastrectomy							**<0.001**
No	2720	(80)	1647	(77)	1073	(86)	
Yes	659	(20)	481	(23)	178	(14)	
Unknown	385		259		126		
Year of diagnosis							**<0.001**
2006–2008	1317	(35)	807	(34)	510	(37)	
2009–2011	1229	(33)	747	(31)	482	(35)	
2012–2014	1218	(32)	833	(35)	385	(28)	
Neoadjuvant treatment							**<0.001**
None	2169	(58)	967	(41)	1202	(87)	
Chemotherapy	1567	(42)	1400	(59)	167	(12)	
Radiotherapy	4	(0.1)	0	(0)	4	(0.3)	
Chemoradiation	24	(0.6)	20	(0.8)	4	(0.3)	
Surgical urgency							**0.014**
(Sub)acute	157	(4)	85	(4)	72	(5)	
Elective	3607	(96)	2302	(96)	1305	(95)	
Surgical type							**<0.001**
Subtotal gastrectomy	2438	(65)	1438	(60)	1000	(73)	
Total gastrectomy	1326	(35)	949	(40)	377	(27)	
Surgical approach							**0.032**
Open	3377	(91)	2120	(90)	1238	(92)	
Laparoscopic	347	(9)	238	(10)	109	(8)	
Unknown	40		29		30		
Radicality							0.948
R0	3164	(87)	2006	(87)	1158	(87)	
R+	461	(13)	293	(13)	168	(13)	
Rx	139		88		51		
(y)pT stage							**<0.001**
T0	118	(3)	102	(4)	16	(1)	
T1	720	(19)	470	(20)	250	(18)	
T2	637	(17)	386	(16)	251	(18)	
T3	1434	(39)	900	(38)	534	(39)	
T4a	811	(22)	494	(21)	317	(23)	
Tx	44		35		9		
(y)pN stage							0.729
N0	1856	(49)	1173	(49)	683	(50)	
N1	689	(18)	440	(18)	249	(18)	
N2	604	(16)	389	(16)	215	(16)	
N3	615	(16)	395	(16)	230	(17)	
Tumor differentiation							**<0.001**
Well	100	(4)	53	(3)	47	(4)	
Moderate	722	(27)	366	(23)	356	(32)	
Poor	1886	(70)	1165	(74)	721	(64)	
Undifferentiated	1	(<0.1)	1	(<0.1)	0	(0)	
Unknown	1055		802		253		
Adjuvant therapy							**<0.001**
No	2736	(73)	1433	(60)	1303	(95)	
Chemotherapy	790	(21)	740	(31)	50	(4)	
Chemoradiation	238	(6)	214	(9)	24	(2)	

Bold values indicate significance (*p* < 0.05). Values were rounded to the nearest percentage point

*SD* standard deviation

### Lymph Node Yield

In young patients, the median LNY was 14 (interquartile range [IQR] 8–22), compared with 11 (IQR 6–18) in elderly patients (*p* < 0.001). In the elderly patients, 851 (62%) had a low LNY, 333 (24%) had an intermediate LNY, and 174 (13%) had a high LNY; no data on LNY were available for 19 (1%) patients. In the young patients, 1180 (49%) had a low LNY, 707 (30%) had an intermediate LNY, and 462 (19%) had a high LNY; no data were available for 38 (2%) patients. Comparison of patient- and treatment-related characteristics between the different LNY groups are presented in Table 2.

**Table 2:** Baseline characteristics regarding the extent of lymph node yield stratified for age (<75 years and ≥75 years)

	Young (<75 years)							Elderly (≥75 years)						
	<15 nodes		15–25 nodes		>25 nodes			<15 nodes		15–25 nodes		>25 nodes		
	*n* = 118)	(%)	*n* = 707	(%)	*n* = 462	(%)	*p* value	*n* = 851	(%)	*n* = 333	(%)	*n* = 174	(%)	*p* value
Age, years (mean [SD])	62.9	[± 9.6]	61.8	[± 10.3]	61.3	[± 10.0]	**0.004**	80.2	[± 3.8]	79.8	[± 3.7]	79.5	[± 3.2]	**0.041**
Sex							0.254							**0.017**
Male	744	(63)	449	(64)	273	(59)		524	(62)	194	(58)	87	(50)	
Female	436	(37)	258	(36)	189	(41)		327	(38)	139	(42)	87	(50)	
Malignancy history							0.081							0.340
No	1042	(88)	643	(91)	422	(91)		686	(81)	275	(83)	148	(85)	
Yes	138	(12)	64	(9)	40	(9)		165	(14)	58	(17)	26	(15)	
Referral for gastrectomy							**<0.001**							**<0.001**
No	977	(86)	422	(71)	217	(60)		736	(91)	224	(79)	94	(67)	
Yes	154	(14)	176	(29)	144	(40)		73	(9)	59	(21)	46	(33)	
Unknown	49		109		101			42		50		34		
Year of diagnosis							**<0.001**							**<0.001**
2006–2008	525	(44)	181	(26)	68	(15)		387	(45)	85	(26)	20	(12)	
2009–2011	408	(35)	211	(30)	124	(27)		313	(37)	115	(34)	53	(30)	
2012–2014	247	(21)	315	(45)	270	(58)		151	(18)	133	(40)	101	(58)	
Neoadjuvant treatment							**<0.001**							**0.008**
None	581	(49)	234	(33)	130	(28)		764	(90)	278	(83)	142	(82)	
Chemotherapy	591	(50)	465	(66)	329	(71)		82	(10)	52	(16)	32	(18)	
Radiotherapy	0	(1)	0	(1)	0	(1)		2	(0.2)	2	(0.6)	0	(0)	
Chemoradiation	8		8		3			3	(0.4)	1	(0.3)	0	(0)	
Surgical urgency							**0.001**							0.095
(Sub)acute	56	(5)	19	(3)	6	(1)		52	(6)	15	(5)	4	(3)	
Elective	1124	(95)	688	(97)	456	(99)		799	(94)	318	(95)	170	(97)	
Surgical type							**<0.001**							**0.001**
Subtotal gastrectomy	817	(69)	375	(53)	226	(49)		647	(76)	223	(67)	115	(66)	
Total gastrectomy	363	(31)	332	(47)	236	(51)		204	(24)	110	(33)	59	(33)	
Surgical approach							**<0.001**							**<0.001**
Open	1110	(95)	599	(86)	373	(83)		795	(95)	295	(89)	138	(81)	
Laparoscopic	58	(5)	101	(14)	79	(18)		41	(5)	36	(11)	32	(19)	
Unknown	12		7		10			15		2		4		
Radicality							0.628							0.076
R0	996	(88)	592	(87)	392	(88)		716	(88)	274	(84)	153	(90)	
R+	138	(12)	92	(13)	52	(12)		97	(12)	53	(16)	17	(10)	
Rx	46		23		18			38		6		4		
(y)pT stage							**0.026**							**<0.001**
T0	47	(4)	34	(5)	18	(4)		8	(1)	7	(2)	1	(0.6)	
T1	265	(23)	124	(18)	77	(17)		194	(23)	29	(9)	24	(14)	
T2	198	(17)	118	(17)	63	(14)		163	(19)	58	(18)	24	(14)	
T3	423	(36)	264	(38)	201	(44)		306	(36)	138	(42)	83	(48)	
T4a	232	(20)	151	(22)	100	(22)		173	(21)	99	(30)	42	(24)	
Tx	15		16		3			7		2		0		
(y)pN stage							**<0.001**							**<0.001**
N0	643	(54)	324	(46)	183	(40)		475	(56)	117	(35)	82	(47)	
N1	247	(21)	117	(17)	70	(15)		162	(19)	64	(19)	18	(10)	
N2	211	(18)	100	(14)	70	(15)		144	(17)	46	(14)	21	(12)	
N3	79	(7)	166	(23)	139	(30)		70	(8)	106	(32)	51	(31)	
Tumor differentiation							**<0.001**							0.145
Well	35	(4)	8	(2)	9	(3)		35	(5)	9	(3)	3	(2)	
Moderate	217	(27)	98	(21)	48	(16)		230	(33)	83	(29)	38	(28)	
Poor	548	(68)	356	(77)	251	(81)		423	(62)	193	(68)	93	(69)	
Undifferentiated	1	(0.1)	0	(0)	0	(0)		0	(0)	0	(0)	0	(0)	
Unknown	379		245		154			163		48		40		
Adjuvant therapy							**<0.001**							0.172
No	784	(66)	387	(55)	235	(51)		812	(95)	308	(92)	164	(94)	
Chemotherapy	314	(27)	245	(35)	171	(37)		29	(3)	16	(5)	5	(3)	
Chemoradiation	82	(7)	75	(11)	56	(12)		10	(1)	9	(3)	5	(3)	

Bold values indicate significance (*p* < 0.05). Values were rounded to the nearest percentage point

*SD* standard deviation

### Postoperative Mortality

The 30- and 90-day mortality in the total study population was 5 and 8%, respectively, and both the 30- and 90-day mortality rates were higher in elderly patients compared with young patients (30-day mortality 10 vs. 3%, *p* < 0.001; 90-day mortality 14 vs. 5%, *p* < 0.001, respectively). In elderly patients, the 30-day mortality within the low (<15), intermediate (15–25), and high (>25) LNY groups was 10, 10, and 8%, respectively, whereas the 90-day mortality was 15, 14, and 10%, respectively. Multivariable analysis did not demonstrate an association between LNY and postoperative mortality for both young and elderly patients (*p* > 0.25) (Table 3).

**Table 3:** Univariable and multivariable logistic regression analyses on the influence of lymph node retrieval on 30-and 90-day mortality in patients treated with gastrectomy for cancer, stratified for age (<75years and ≥75 years)

Young (<75 years)	30-day mortality						90-day mortality					
	Univariable			Multivariable^a^			Univariable			Multivariable^a^		
	OR	95% CI	*p* value	OR	95% CI	*p* value	OR	95% CI	*p* value	OR	95% CI	*p* value
Each additional node	0.99	0.97–1.02	0.445	0.99	0.96–1.02	0.521	0.99	0.97–1.01	0.183	0.99	0.96–1.01	0.298
<15 nodes	Ref	–	**–**	Ref	–	–	Ref	–	–	Ref	–	–
15–25 nodes	0.72	0.40–1.30	0.269	0.65	0.32–1.32	0.236	0.71	0.45–1.11	0.134	0.81	0.47–1.37	0.429
>25 nodes	0.82	0.43–1.59	0.565	0.85	0.36–1.99	0.701	0.74	0.44–1.24	0.250	0.74	0.37–1.48	0.400

Elderly (≥75 years)	30-day mortality						90-day mortality					
	Univariable			Multivariable^a^			Univariable			Multivariable^a^		
	OR	95% CI	*p* value	OR	95% CI	*p* value	OR	95% CI	*p* value	OR	95% CI	*p* value
Each additional node	0.99	0.97–1.01	0.173	1.00	0.98–1.03	0.940	0.99	0.97–1.01	0.173	0.99	0.97–1.02	0.573
<15 nodes	Ref	–	–	Ref	–	–	Ref	–	–	Ref	–	–
15–25 nodes	0.85	0.59–1.22	0.367	0.94	0.56–1.58	0.823	0.85	0.59–1.22	0.367	0.87	0.55–1.36	0.538
>25 nodes	0.66	0.38–1.14	0.132	0.74	0.34–1.60	0.442	0.66	0.38–1.14	0.132	0.63	0.32–1.23	0.178

^a^Adjusted for age, sex, malignancy history, referral, year of diagnosis, tumor differentiation, neoadjuvant therapy, surgical urgency, type of surgery, surgical approach, radicality, (y)pT stage, and (y)pN stage

*OR* odds ratio, *CI* confidence interval

### Overall Survival

**Fig. 1:**
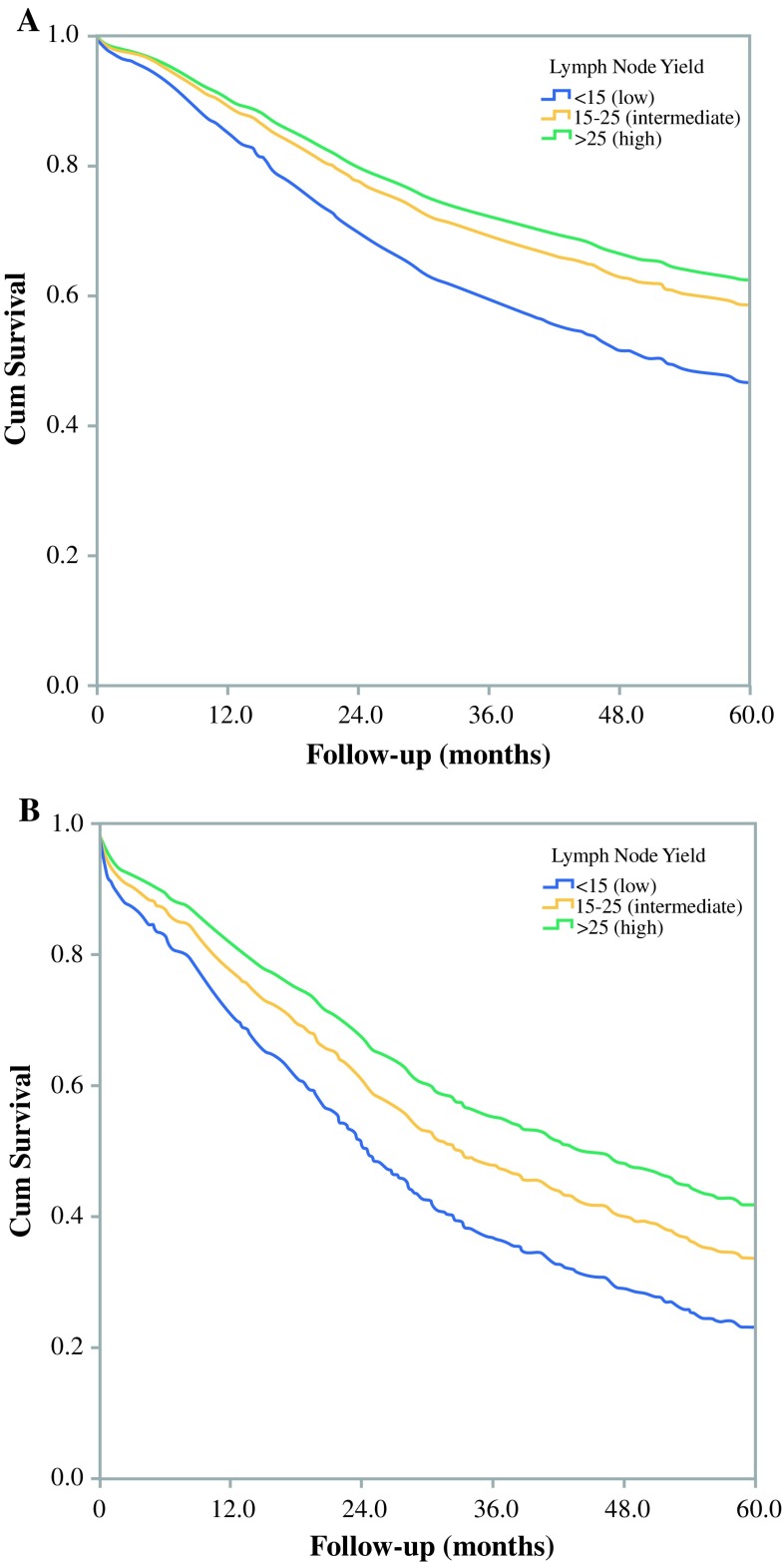
Adjusted survival curves from the proportional hazards model of the 5-year overall survival of **a** young patients (<75 years) and **b** elderly patients (≥75 years), stratified for lymph node yield (<15 nodes, 15–25 nodes and >25 nodes). *Cum* Cumulative

The median overall survival of all patients was 41 months; 58 months in young patients compared with 27 months in elderly patients. The median survival of elderly patients in the low (<15), intermediate (15–25), and high (>25) LNY groups was 26, 26, and 31 months, respectively (*p* = 0.228), whereas the median survival of young patients was 61, 58, and 52 months, respectively (*p* = 0.482). In multivariable analysis, a higher LNY in elderly patients was significantly associated with a prolonged survival when analyzed as a discrete variable (each additional node: HR 0.98, 95% CI 0.97–0.99, *p* < 0.001) or a categorical variable (<15 nodes: reference; 15–25 nodes: HR 0.74, 95% CI 0.62–0.88, *p* < 0.001; >25 nodes: HR 0.59, 95% CI 0.45–0.78, *p* < 0.001). These results were comparable for young patients (Table 4). Figure 1 shows the adjusted survival curves of elderly and young patients, stratified by LNY. Subgroup analysis demonstrated that R status did not influence the association between LNY and overall survival (data not shown).

**Table 4:** Univariable and multivariable Cox regression analyses on the influence of lymph node retrieval on overall survival in patients treated with gastrectomy for cancer, stratified for age (<75 years and ≥75 years)

	Young (<75 years)						Elderly (≥75 years)					
	Univariable			Multivariable^a^			Univariable			Multivariable^a^		
	HR	95% CI	*p* value	HR	95% CI	*p* value	HR	95% CI	*p* value	HR	95% CI	*p* value
Lymph node yield												
<15 nodes	Ref	–	**–**	Ref	–	**–**	Ref	–	**–**	Ref	–	**–**
15–25 nodes	0.97	0.85–1.11	0.68	0.71	0.60–0.83	**<0.001**	1.03	0.88–1.21	0.69	0.75	0.63–0.89	**0.001**
>25 nodes	1.07	0.91–1.26	0.41	0.62	0.51–0.76	**<0.001**	0.83	0.67–1.04	0.10	0.61	0.47–0.80	**<0.001**
Additional year of age	1.01	1.00–1.02	**0.001**	1.01	1.00–1.02	**0.008**	1.04	1.03–1.06	**<0.001**	1.03	1.02–1.05	**<0.001**
Sex												
Male	Ref	–	**–**	Ref	–	**–**	Ref	–	**–**	Ref	–	**–**
Female	1.001	0.89–1.13	0.982	1.07	0.94–1.22	0.325	0.91	0.80–1.04	0.180	0.80	0.69–0.92	**0.002**
Malignancy history												
No history of malignancy	Ref	–	**–**	Ref	–	**–**	Ref	–	**–**	Ref	–	**–**
Malignancy in history	1.21	1.01–1.45	**0.043**	1.20	0.98–1.46	0.076	1.21	1.03–1.42	**0.021**	1.38	1.15–1.64	**<0.001**
Referral												
Same hospital	Ref	–	**–**	Ref	–	**–**	Ref	–	**–**	Ref	–	**–**
Other hospital	0.98	0.85–1.14	0.812	0.99	0.83–1.17	0.859	1.04	0.85–1.26	0.730	1.23	0.99–1.54	0.064
Additional year of diagnosis	1.00	0.97–1.02	0.790	1.06	1.02–1.09	**0.002**	0.96	0.93–0.99	**0.003**	0.98	0.95–1.02	0.360
Tumor differentiation												
Well	Ref	–	**–**	Ref	–	**–**	Ref	–	**–**	Ref	–	**–**
Moderate	1.88	1.12–3.13	**0.016**	0.97	0.57–1.65	0.897	1.07	0.72–0.60	0.739	0.84	0.55–1.28	0.415
Poor	2.31	1.41–3.80	**0.001**	0.95	0.56–1.59	0.831	1.73	1.18–2.55	**0.006**	1.07	0.71–1.61	0.756
Undifferentiated	3.95	0.52–29.8	0.182	0.87	0.51–1.48	0.608	NA	NA	NA	NA	NA	NA
Neoadjuvant therapy												
None	Ref	–	**–**	Ref	–	**–**	Ref	–	**–**	Ref	–	**–**
Chemotherapy	0.83	0.74–0.93	**0.001**	1.10	0.93–1.29	0.264	0.63	0.50–0.79	**<0.001**	0.94	0.69–1.27	0.682
Chemoradiotherapy	0.78	0.39–1.57	0.485	0.42	0.14–1.32	0.139	2.49	0.93–6.65	0.070	2.92	0.91–1.46	0.072
Surgical urgency												
Elective	Ref	–	**–**	Ref	–	**–**	Ref	–	**–**	Ref	–	**–**
(Sub)acute	1.44	1.09–1.90	**0.011**	1.16	0.85–1.58	0.342	1.22	0.92–1.60	0.168	1.11	0.82–1.50	0.501
Type of surgery												
Partial	Ref	–	**–**	Ref	–	**–**	Ref	–	**–**	Ref	–	**–**
Total	1.41	1.26–1.59	**<0.001**	1.27	1.11–1.45	**<0.001**	1.29	1.12–1.49	**<0.001**	1.25	1.07–1.46	**0.005**
Surgical approach												
Open	Ref	–	**–**	Ref	–	**–**	Ref	–	**–**	Ref	–	**–**
Minimally invasive	0.93	0.73–1.18	0.54	0.98	0.72–1.33	0.901	0.88	0.65–1.18	0.391	0.96	0.64–1.45	0.849
Radicality												
R0	Ref	–	**–**	Ref	–	**–**	Ref	–	**–**	Ref	–	**–**
R1	3.18	2.74–3.70	**<0.001**	1.65	1.39–1.96	**<0.001**	2.38	1.99–2.84	**<0.001**	0.45	1.18–1.77	**<0.001**
R2	6.60	4.13–10.5	**<0.001**	1.95	1.11–3.45	**0.021**	3.98	2.24–7.05	**<0.001**	2.32	1.20–4.48	**0.012**
(y)pT stage												
T0	Ref	–	**–**	Ref	–	**–**	Ref	–	**–**	Ref	–	**–**
T1	1.17	0.67–2.06	0.579	1.07	0.57–2.02	0.839	1.22	0.50–3.00	0.655	0.91	0.32–2.60	0.854
T2	2.45	1.42–4.25	**0.001**	1.80	1.97–3.36	0.064	1.63	0.67–3.97	0.258	1.09	0.38–3.10	0.872
T3	5.34	3.14–9.08	**<0.001**	3.20	1.74–5.87	**<0.001**	3.03	1.25–7.32	**0.014**	1.57	0.56–4.43	0.396
T4a	7.65	4.48–13.0	**<0.001**	3.68	1.96–6.81	**<0.001**	4.19	1.73–10.1	**0.002**	1.85	0.65–5.26	0.249
(y)pN stage												
N0	Ref	–	**–**	Ref	–	**–**	Ref	–	**–**	Ref	–	**–**
N1	2.29	1.94–2.69	**<0.001**	1.71	1.43–2.06	**<0.001**	1.86	1.58–2.56	**<0.001**	1.60	1.31–1.96	**<0.001**
N2	3.28	2.79–3.85	**<0.001**	2.43	2.02–2.92	**<0.001**	2.54	2.11–3.05	**<0.001**	2.10	1.71–2.58	**<0.001**
N3a	4.82	4.06–5.74	**<0.001**	3.65	2.97–4.48	**<0.001**	4.18	3.46–5.05	**<0.001**	3.26	2.59–4.10	**<0.001**
N3b	8.99	8.99–7.10	**<0.001**	6.38	4.74–8.59	**<0.001**	5.02	3.65–6.90	**<0.001**	4.81	3.27–7.09	**<0.001**
Adjuvant therapy												
None	Ref	–	**–**	Ref	–	**–**	Ref	–	**–**	Ref	–	**–**
Chemotherapy	0.71	0.62–0.81	**<0.001**	0.67	0.56–0.79	**<0.001**	0.53	0.35–0.82	**0.005**	0.73	0.43–1.23	0.241
Chemoradiotherapy	0.94	0.77–1.16	0.575	0.70	0.56–0.88	**0.002**	0.94	0.57–1.57	0.825	0.43	0.24–0.78	**0.005**

Bold values indicate significance (*p* < 0.05)

*HR* hazard ratio, *CI* confidence interval, *NA* not applicable

## Discussion

This population-based observational cohort study aimed to evaluate the influence of LNY on postoperative mortality and overall survival in patients aged 75 years or older who underwent curative gastrectomy for cancer. The results indicate that a high LNY improves survival for both young and elderly patients. In addition, LNY was not associated with postoperative mortality in these patients.

The findings of the present study are in line with several large population-based studies investigating the effect of LNY on survival.^15^,^16^ One of these studies found a linear trend for improved survival based on more harvested nodes, up to a cut-off point of 40 lymph nodes.^15^ Moreover, other studies demonstrated that in patients with a low LNY, no adequate prediction of patient survival could be made, suggesting inadequate staging.^17^,^18^ International and national guidelines therefore require examination of ≥15 lymph nodes for adequate staging of gastric cancer.^9^,^14^

As elderly form a substantial portion of gastric cancer patients^9^ and are prone to postoperative morbidity and mortality,^6^ the extent of lymphadenectomy in elderly patients with gastric cancer has been under debate. Recently, a French study did not find a difference in survival between a high, low, and intermediate LNY in patients aged 75 years and older who underwent curative gastrectomy for cancer.^10^ Even though a higher LNY did not affect postoperative morbidity and mortality, the authors advise a limited lymphadenectomy due to the lack of a survival benefit. However, the effect of LNY on the outcomes was assessed in univariable analysis only, which does not allow for correction of well-known confounders such as patient and tumor characteristics. Such confounders may introduce bias, which is a challenging problem in retrospective observational studies.^19^ These limitations, as well as the possible implications of the authors’ recommendations on daily practice, made evaluation of these findings warranted. The present study has taken these limitations into account by including almost four times as many elderly patients from a more recent cohort, and by performing a multivariable analysis. The current results confirm the absence of increased postoperative mortality, but support the oncological value of an extensive lymphadenectomy in all patients undergoing curative gastrectomy for cancer with a clear survival benefit.

The present study chose a cut-off point of 75 years to be able to make a fair comparison with the French study; however, the definition of ‘elderly’ is arbitrary and we do not believe that age should be a ‘hard-stop’ discriminator for the choice of the extent of treatment. In our opinion, patient fitness rather than age should be considered when choosing the appropriate treatment. For patient fitness, factors such as comorbidities, smoking status, and weight loss should be taken into account.^20^

Interestingly, LNY increased over the years, especially in the last period (2012–2014). In The Netherlands, a D2 lymphadenectomy has been standard of care since the final publication of the Dutch D1–D2 trial;^21^ therefore, a D2 lymphadenectomy was recommended throughout the whole study period. It could be that surgical quality increased over the years due to centralization of stomach surgery in The Netherlands (started in 2009), but no data are currently available to support this hypothesis. Another factor is the start of the Dutch Upper GI Cancer Audit (DUCA) in 2011, which has contributed to an increase in LNY.^22^ In the DUCA, LNY (>15 nodes) is seen as an important quality indicator, which may have motivated centers to increase their LNY.^22^ This increase may then be due to an improvement in surgical quality, but could also be attributed to a more thorough inspection of the resection specimen by pathologists.

It is important to realize that LNY does not fully correspond to the extent of lymphadenectomy. Although a D2 lymphadenectomy is recommended by national and international guidelines,^11^,^23^ the Dutch D1–D2 trial demonstrated that more than half of the resection specimens, which were indicated to have had a D2 lymphadenectomy, lacked two or more of the required lymph node stations.^24^ On the other hand, one-quarter of these resection specimens included more than the intended lymph nodes stations. These results indicate that both the French study and the current study do not know exactly which lymphadenectomy was actually performed by the surgeons. In addition to these uncertainties, there might be variation in lymphadenectomy between surgeons, variation in the submission of specimens (*en bloc* or in separate containers),^25^ and variation in lymph node retrieval by pathologists,^26^ all of which may influence the final LNY. All these factors combined imply that caution should be taken in drawing conclusions on the extent of lymphadenectomy, based solely on counting lymph nodes. In addition, the applicability of LNY as a surrogate for lymphadenectomy without data on tumor recurrence should be carefully interpreted. Ideally, a randomized controlled trial comparing different types of lymphadenectomies in elderly patients should be performed in order to provide a fair answer to this topic. On the other hand, such a study might not be considered ethical regarding the favorable results of an extensive lymphadenectomy,^7^,^27^ especially since postoperative mortality seems not to be increased as a result of the lymphadenectomy.

Although the present study corrected for many confounding factors, including patient and tumor characteristics, data on patients’ comorbidities, American Society of Anesthesiologists (ASA) status, body mass index (BMI), and disease recurrence were not available from the NCR database. Therefore, we could not correct for these well-known patient confounders, which could have influenced the extent of lymphadenectomy performed (selection bias), nor could we investigate the influence of LNY on disease-free survival. For instance, patients with poor performance status or severe comorbidities might have undergone a less extended lymphadenectomy. Moreover, NCR data lack information on the type of chemotherapy and the number of cycles, and administration of palliative chemotherapy is absent. Palliative chemotherapy has been shown to improve survival compared with supportive care.^28^ As a large number of patients develop distant metastases, the use of systemic therapy may influence survival. Furthermore, no data on hospital volume were available, which may influence both LNY and postoperative outcomes. Lastly, there might be some unknown confounding due to the retrospective nature of the study.

## Conclusion

A high LNY is associated with prolonged survival but not with an increase in postoperative mortality, for young as well as elderly patients. Therefore, an extensive lymphadenectomy cannot be abandoned as the preferred strategy and should be considered in all patients during gastrectomy.
